# Addition of hydrochloric acid to collection bags or collection containers did not change basal endogenous losses or ileal digestibility of amino acid in corn, soybean meal, or wheat middlings fed to growing pigs

**DOI:** 10.5713/ab.20.0838

**Published:** 2021-03-02

**Authors:** Su A Lee, Laia Blavi, Diego M. D. L. Navarro, Hans H. Stein

**Affiliations:** 1 Department of Animal Sciences, University of Illinois, Urbana, IL 61801, USA; 2 Departament de Ciència Animal i dels Aliments, Universitat Autònoma de Barcelona, Barcelona, Spain; 3 Hamlet Protein Inc., Findlay, OH 45840, USA

**Keywords:** Amino Acids, Collection, Digestibility, Hydrogen Chloride, Ileal Digesta, Pigs

## Abstract

**Objective:**

The hypothesis was that apparent ileal digestibility (AID), basal endogenous losses, and standardized ileal digestibility (SID) of amino acids (AA) are not affected by adding acid to collection containers or bags used to collect ileal digesta from pigs.

**Methods:**

Twenty-four growing barrows (initial body weight: 77.8±4.5 kg) that were fitted with a T-cannula in the distal ileum were fed diets for three 7-d periods. An N-free diet and 3 diets containing corn, soybean meal, or wheat middlings as the sole source of AA were used. Within each period, each of the 4 diets were fed to 6 pigs. Among the 6 pigs, digesta from 3 pigs were collected in bags containing no HCl, whereas 40 mL of 3 *N* HCl was included in the bags used to collect digesta from the remaining 3 pigs. Every other bag collected from each pig was emptied into a container without adding HCl, whereas the remaining bags were added to a container along with 40 mL of 3 *N* HCl for each bag. All digesta were stored at −20°C immediately after collection. Data were analyzed using a model that included feed ingredient, HCl in bags, HCl in containers, and all 2-way and 3-way interactions as fixed effects. No 3-way interactions were significant, and data were, therefore, reanalyzed independently for each diet as a 2×2 factorial.

**Results:**

There were no interactions between adding HCl to collection bags and to containers, and no effects of adding HCl to collection bags or containers for AID, basal endogenous losses, or SID of most AA were observed.

**Conclusion:**

It is not necessary to add acid to digesta collection bags or collection containers if ileal digesta are stored at −20°C immediately after collection.

## INTRODUCTION

Dietary protein is digested in the small intestine of pigs and free amino acids (AA) or small peptides are absorbed before the end of the small intestine. Therefore, ileal digestibility is the most accurate estimation of AA digestibility because by measuring ileal digestibility interference by microbes in the hind-gut is avoided [1]. However, there is also microbial fermentation in the small intestine of pigs [2], and ileal digesta, therefore, contain microbial protein [3]. Therefore, AA in ileal digesta may be metabolized unless microbial action is stopped immediately after collection.

In most AA digestibility experiments collection bags are changed at least every 30 min and the collected ileal digesta are stored at −20°C to prevent microbial degradation of N. Another attempt to stop microbial action in the digesta includes adding acids to collection bags [4,5] or to collection containers [6,7] to reduce pH of ileal digesta. To our knowledge, there is, however, no information about the need for adding acids to collected digesta and it is not known if values for apparent ileal digestibility (AID), basal endogenous losses of AA, or standardized ileal digestibility (SID) of AA are influenced by addition of acids to digesta. Therefore, the objective of this experiment was to test the null-hypothesis that values for AID, basal endogenous losses, and SID of AA by pigs are not influenced by the addition of acids to ileal digesta collection bags or to collection containers regardless of the ingredient being fed.

## MATERIALS AND METHODS

The Institutional Animal Care and Use Committee at the University of Illinois reviewed and approved the protocol for the experiment. Pigs used in the experiment were the offspring of L 359 boars mated to Camborough females (Pig Improvement Company, Hendersonville, TN, USA).

### Animals, experimental design, housing, and feeding

Twenty-four barrows (initial body weight: 77.8±4.5 kg) that were equipped with a T-cannula in the distal ileum [8] were used. Pigs had been used in a different experiment before being fed a common finisher diet for 14 d and then used in this experiment. Pigs were allotted to 4 diets with 6 pigs being fed each of the 4 diets in each of the 3 periods. Pigs were housed in individual pens (1.2×1.5 m) and each pen was equipped with a feeder and a nipple drinker and had fully slatted tribar floors and smooth sidewalls.

Pigs were fed their assigned diets in a daily amount of 3.4 times the estimated energy requirement for maintenance (i.e., 197 kcal metabolizable energy per kg^0.60^) [9], and 2 equal meals were provided every day at 0800 and 1600 h. Water was available at all times.

### Treatments

A total of 4 diets were formulated (Table 1). Three diets contained corn, soybean meal (SBM), or wheat middlings as the only source of protein and AA, and the fourth diet was an N-free diet that was used to determine endogenous losses of AA (Tables 2, 3). Vitamins and minerals were included in all diets to meet or exceed estimated nutrient requirements for growing pigs [9]. All diets also contained 0.40% chromic oxide as an indigestible marker. All diets were fed to pigs in mash form.

The 24 pigs were allotted to 4 diets and 3 periods using the balanced Latin square design program [10]. Thus, 6 pigs were fed each diet in each period. Among the 6 pigs fed the same diet, digesta from 3 pigs were collected in bags containing no HCl whereas 40 mL of 3 N HCl was included in the bags used to collect digesta from the remaining 3 pigs. Following collection, digesta bags were alternately emptied into collection containers without adding HCl or into containers along with 40 mL of 3 *N* HCl for each bag. All containers were stored at −20°C at all times. Therefore, for pigs fed the 3 diets containing corn, SBM, and wheat middlings, there were a total of 12 treatments arranged in a 3×2×2 factorial with feed ingredients (i.e., corn, SBM, and wheat middlings) as the first factor and no HCl or addition of HCl in either collection bags or collection containers as the second and third factors, respectively. For pigs fed the N-free diet, there were a total of 4 treatments that were arranged in a 2×2 factorial with no HCl or addition of HCl in either collection bags or collection containers. Three replicate pigs were used per treatment in each period and there were a total of 9 replications per treatment for the 3 periods.

### Sample collection

Pig weights were recorded at the beginning of each period and at the conclusion of the experiment and the amount of feed supplied each day was recorded. The initial 5 d of each period was considered an adaptation period to the diets. Ileal digesta were collected for 8 h (from 0800 to 1600 h) on d 6 and 7 by attaching a plastic bag to the opened cannula barrel, and digesta flowing into the bag were collected and stored in collection containers at −20°C. Bags were removed whenever they were filled with digesta or at least once every 30 min. On the completion of one experimental period, animals were deprived of feed overnight and the following morning, a new experimental diet was offered.

At the conclusion of the experiment, all containers were removed from freezers, the ileal digesta were thawed and mixed in each container, and a sub-sample was collected for chemical analysis. Ileal digesta samples were lyophilized and finely ground prior to analysis.

### Chemical analysis

Based on the methods described in AOAC Int. [11], corn, SBM, wheat middlings, diets, and ileal digesta samples were analyzed for dry matter (method 930.15) and ingredients were analyzed for ash (method 942.05). Ingredient samples were also analyzed for insoluble and soluble dietary fiber using the Ankom Dietary Fiber Analyzer (Ankom Technology, Macedon, NY, USA; method 991.43). Total dietary fiber was calculated as the sum of soluble and insoluble dietary fiber. Acid hydrolyzed ether extract in corn, SBM, and wheat middlings was analyzed by acid hydrolysis using 3 *N* HCl (Ankom^HCl^, Ankom Technology, USA) followed by crude fat extraction using petroleum ether (Ankom^XT15^, Ankom Technology, USA). Ingredients and diets samples were analyzed for N by combustion (method 999.03) [11] on an Elementar Rapid N-cube protein/nitrogen apparatus (Elementar Americas Inc., Mt. Laurel, NJ, USA). Aspartic acid was used as a calibration standard and crude protein was calculated as N×6.25. These samples were also analyzed for AA on a Hitachi Amino Acid Analyzer (Model L8800, Hitachi High Technologies America Inc., Pleasanton, CA, USA) using ninhydrin for post-column derivatization and nor-leucine as the internal standard. Samples were hydrolyzed with 6 *N* HCl for 24 h at 110°C prior to analysis, but Met and Cys were analyzed as methionine sulfone and cysteic acid after cold performic acid oxidation overnight before hydrolysis, and Trp was determined after NaOH hydrolysis for 22 h at 110°C (method 982.30 E [a, b, c]) [11]. Ingredients and diets were analyzed for gross energy using an isoperibol bomb calorimeter (Model 6400, Parr Instruments, Moline, IL, USA). Benzoic acid was used as the standard for calibration. The chromium concentration in diets and ileal digesta samples was determined using Inductive Coupled Plasma Atomic Emission Spectrometric method (method 990.08) [11]. Samples were prepared using nitric acid-perchloric acid (method 968.08D[b]) [11].

### Calculations and statistical analysis

Values for the AID, basal endogenous losses, and SID of AA in each diet were calculated as described previously [12]. Four values for basal endogenous losses of AA were determined: from samples stored without or with HCl in collection bags and without or with HCl in collection containers. Values for SID of AA were calculated by correcting AID values for each treatment for basal endogenous losses calculated for the same treatment. Therefore, for corn, SBM, and wheat middlings, there were 4 AID and SID values calculated based on the inclusion or not of HCl in collection bags and inclusion or not of HCl in collection containers.

Normality of residuals was verified using the UNIVARIATE procedure (SAS Inst. Inc., Cary, NC, USA). Outliers were identified as values that deviated from the treatment mean by more than 3 times the interquartile range [13]. Data were analyzed using a split-plot arrangement in PROC MIXED (SAS Inst. Inc., USA) with the 3 feed ingredients and the 2 levels of HCl in collection bags as whole-plot factors, and the 2 levels of HCl in collection containers as the split-plot factor. The initial model included the fixed effects of feed ingredients, HCl in collection bags, HCl in collection containers, all 2-way interactions, and all 3-way interactions. The random effects were HCl in collection bags and square, period, and pig nested within HCl in collection bags. However, no 3-way interactions were significant (p>0.10), and data were, therefore, analyzed independently for each ingredient as a 2×2 factorial using the same split-plot arrangement as used for the initial model. Data for basal endogenous losses were also analyzed using this model. Treatment means were calculated by using the LSMEANS statement and if the interaction was significant, the PDIFF option was used to separate means. Statistical significance and tendency were considered at p< 0.05 and 0.05≤p<0.10, respectively.

## RESULTS

Pigs remained healthy during the experiment and very little feed refusals were observed.

### Apparent ileal digestibility of amino acids

For corn, SBM, and wheat middlings, there were no interactions between HCl in collection bags and in collection containers for the AID of any AA (Tables 4, 5, and 6). No effects of adding HCl to collection bags or collection containers for the AID of AA in corn, SBM, or wheat middlings were observed with the exception that the AID of Met in wheat middlings was greater (p = 0.049) if HCl was added to collection bags compared with no HCl in the bags. The AID of Met in SBM tended to be greater (p = 0.060) if HCl was added to collection containers than if no HCl was added. The AID of Phe in wheat middlings tended to be greater (p = 0.085) if HCl was added to collection bags than if no HCl was added. The AID of total dispensable AA in wheat middlings was also greater (p<0.05) if HCl was added to collection containers than if no HCl was added.

### Basal endogenous losses of amino acids

No interactions between the addition of HCl to collection bags and collection containers were observed for basal endogenous losses of AA (Table 7). There were also no effects of adding HCl to either collection bags or collection containers.

### Standardized ileal digestibility of amino acids

No interactions between adding HCl to collection bags and containers for the SID of AA in corn were observed, and there were no effects of adding HCl to either collection bags or containers for the SID of AA in corn (Table 8). For SBM, no interactions between adding HCl to collection bags and containers for the SID of AA except for Arg, Gly, and Ser were observed (Table 9). Addition of HCl only to collection containers decreased (p<0.05) the SID of Arg and Ser in SBM, but no HCl effect was detected among other treatments (interaction; p<0.05). The SID of Gly was less (p<0.05) if HCl was added only to collection containers than if HCl was added to collection bags and containers, but no difference was observed among other treatments (interaction; p<0.05). Addition of HCl to collection bags did not change values for SID of most AA in SBM and there were no effects of adding HCl to collection containers with the exception that the SID of Met was greater (p = 0.041) if HCl was added to collection containers than if no HCl was added to containers. For the SID of AA in wheat middlings, no interactions between adding HCl to collection bags and containers were observed (Table 10). Adding HCl to either collection bags or collection containers did not affect the SID of AA in wheat middlings.

## DISCUSSION

The direct procedure was used to determine SID of AA in corn, SBM, and wheat middlings although this procedure results in diets based on corn and wheat middlings having AA concentrations below the requirement for the pigs. However, feeding diets with AA below the requirement does not influence the SID of AA [14]. The three ingredients were chosen because they have different concentrations of fiber and protein and therefore may differently affect ileal digesta characteristics. Concentrations of AA in corn, SBM, and wheat middlings, values for the SID of AA in corn and SBM, and the basal endogenous losses of AA were reasonably close to values in the literature [9,15]. The SID of most AA in wheat middlings was in agreement with previous data [16], but the SID of Lys in wheat middlings was greater than reported [16]. Lysine is usually the AA that is most susceptible to heat damage that may take place during production because Lys has an amino group in the side chain [17]. The fact that the SID of Lys in wheat middlings used in this experiment was greater compared with previous values indicates that heat damage during processing was minimal. Greater standard error of the means values for the AID and SID of AA were observed in wheat middlings compared with corn and SBM. Wheat middlings contain more fiber than corn and SBM, and it is possible that greater concentration of fiber increases variations in digestibility values. Recovery of the indigestible marker can also be affected by fiber [18], which may result in greater variations.

Dietary protein is digested and absorbed as free AA or small peptides in the small intestine of pigs, but undigested and unabsorbed N and AA are fermented in the hind-gut. Addition of antibiotics to diets fed to pigs reduces N fermentation [19], which indicates that prevention of microbial fermentation in ileal digesta also prevents the change in AA composition of the ileal digesta of pigs. Because AA are degraded, synthesized, or both degraded and synthesized by microbial fermentation [12], it was expected that depending of AA, ileal digestibility was increased, decreased, or remained the same if microbial fermentation affects the collected ileal digesta.

Because microbial fermentation also occurs in the small intestine of pigs [2,3,19], stopping microbial action after collection of ileal digesta is necessary. By storing ileal digesta in a −20°C freezer immediately after collection, microbial action can be prevented [20,21]. In most AA digestibility experiments the collected ileal digesta are stored at −20°C to prevent microbial degradation of AA. However, it has been suggested that adding acid to collection bags [4,5] or collection containers [6,7] may reduce pH and, therefore, stop fermentation in the digesta [22,23], which theoretically will result in changed SID of AA. Both formic acid (HCOOH) and HCl have been used in previous experiments whereas only HCl was used in this experiment. Formic acid is less acidic compared with HCl because of the lower acid dissociation constant, which may result in a reduced ability to reduce pH in the ileal digesta. It is, therefore, unlikely that formic acid would produce results that are different from those observed in the present experiment with HCl.

However, in some experiments, no attempt to reduce pH is made because it is assumed that the rapid reduction in temperature after collection that is a consequence of storing digesta at −20°C is sufficient to prevent microbial degradation of AA [24–27]. There are no data demonstrating the necessity of adding acids to digesta collection bags or collection containers and it is unlikely that acids added to collection bags will impact fermentation because no mixing of digesta and acids takes place in the bags. However, if acids are added to collection containers, acids can be mixed with digesta, but the observation that adding HCl to collection containers did not impact digestibility indicates that reduction of pH is not necessary to prevent fermentation. This is in agreement with data indicating that adjustment of pH by adding HCl to thawed ileal digesta before drying did not affect AID of AA in diets [28]. The present results indicating no impact of adding HCl to ileal digesta before freezing, therefore, complement the previous data. The observation that the AID of Met and total dispensable AA was increased by adding HCl to collection bags or containers may be a consequence of the fact that microbes in the intestinal tract prefer peptides or ammonia as N rather than free AA [29]. It is, therefore, possible that microbes produce more Met and dispensable AA, specifically Gly, compared with other AA, which may result in an increase in concentrations of Met and Gly in the ileal digesta with no HCl. However, because there was no impact of acids on the SID of any indispensable AA in wheat middlings, the differences observed for AID of Met is likely not of any consequence in practical diet formulation.

The tendency for an interaction between adding HCl to collection bags and to collection containers that was observed for the basal endogenous losses of Lys and all dispensable AA may be a result of a larger proportion of the AA reaching the hindgut of pigs fed the N-free diet being of microbial origin compared with pigs fed the other diets. Although the majority of microbial action takes place in the large intestine, there is some microbial activity in the small intestine [3], which may impact small intestinal digesta differently if an N-free and low fiber diet is provided rather than a diet containing AA and fiber at greater levels. However, research is needed to verify this hypothesis.

It is possible that changing collection bags at least every 30 min or whenever they are full contributed to the lack of difference observed in this experiment because several hours are needed for degradation of AA by microbes [30–32]. The observation that there were no effects of HCl in collection bags or containers on AID and SID of most AA in corn, SBM, and wheat middlings thus indicates that the rapid reduction in temperature after collection is sufficient to prevent microbial degradation of AA. The observation that endogenous losses of AA were also not affected by acids in collection bags or containers further support the hypothesis that it is not necessary to use HCl in AA digestibility experiments.

## CONCLUSION

If digesta are stored at −20°C immediately after collection, there are no clear advantages of adding HCl to digesta collection bags or collection containers. Values for the basal endogenous losses of AA and the AID and SID of most AA are not changed by HCl and it is, therefore, most practical to avoid adding HCl to collection bags or collection containers.

## Figures and Tables

**Table 1:** Ingredient composition of experimental diets, as-is basis

Ingredient (%)	Corn	Soybean meal	Wheat middlings	N-free
Ground corn	94.90	-	-	-
Soybean meal	-	38.00	-	-
Wheat middlings	-	-	40.00	-
Cornstarch	-	45.70	42.20	67.22
Soybean oil	2.00	3.60	-	4.00
Ground limestone	0.70	0.65	1.15	0.46
Dicalcium phosphate	1.30	0.95	0.55	1.72
Sodium chloride	0.40	0.40	0.40	0.40
Sucrose	-	10.00	15.00	20.00
Chromic oxide	0.40	0.40	0.40	0.40
Magnesium oxide	-	-	-	0.10
Potassium carbonate	-	-	-	0.40
Solka floc^1)^	-	-	-	5.00
Vitamin and mineral premix^2)^	0.30	0.30	0.30	0.30

1) Fiber Sales and Development Corp., Urbana, OH, USA.

2) The vitamin-micromineral premix provided the following quantities of vitamins and micro minerals per kg of complete diet: vitamin A as retinyl acetate, 11,136 IU; vitamin D_3_ as cholecalciferol, 2,208 IU; vitamin E as DL-alpha tocopheryl acetate, 66 IU; vitamin K as menadione dimethylprimidinol bisulfite, 1.42 mg; thiamin as thiamine mononitrate, 0.24 mg; riboflavin, 6.59 mg; pyridoxine as pyridoxine hydrochloride, 0.24 mg; vitamin B_12_, 0.03 mg; D-pantothenic acid as D-calcium pantothenate, 23.5 mg; niacin, 44.1 mg; folic acid, 1.59 mg; biotin, 0.44 mg; Cu, 20 mg as copper sulfate and copper chloride; Fe, 126 mg as ferrous sulfate; I, 1.26 mg as ethylenediamine dihydriodide; Mn, 60.2 mg as manganese sulfate; Se, 0.3 mg as sodium selenite and selenium yeast; and Zn, 125.1 mg as zinc sulfate.

**Table 2:** Analyzed composition of the ingredients, as-is basis

Item (%)	Corn	Soybean meal	Wheat middlings
Dry matter	86.72	89.62	88.89
Gross energy (kcal/kg)	3,808	4,191	4,053
Ash	1.01	7.31	4.79
Total dietary fiber	9.7	16.0	36.9
Soluble dietary fiber	ND	0.8	1.9
Insoluble dietary fiber	9.7	15.2	35.0
Acid hydrolyzed ether extract	3.06	1.27	4.07
Crude protein	7.52	48.00	16.57
Indispensable amino acids			
Arg	0.36	3.43	1.09
His	0.22	1.22	0.44
Ile	0.26	2.13	0.50
Leu	0.87	3.66	1.00
Lys	0.28	3.00	0.73
Met	0.15	0.63	0.23
Phe	0.37	2.45	0.65
Thr	0.27	1.82	0.52
Trp	0.04	0.68	0.17
Val	0.35	2.22	0.74
Dispensable amino acids			
Ala	0.53	2.03	0.77
Asp	0.50	5.17	1.13
Cys	0.16	0.62	0.32
Glu	1.34	8.43	2.88
Gly	0.30	1.99	0.86
Ser	0.35	1.99	0.59
Tyr	0.21	1.77	0.41

ND, not detectable.

**Table 3:** Analyzed composition of the experimental diets

Item (%)	Diet			

	Corn	Soybean meal	Wheat middlings	N-free
Dry matter	89.09	94.23	94.05	96.19
Crude protein	6.90	19.96	6.91	0.40
Gross energy (kcal/kg)	3,807	4,003	3,747	3,895
Indispensable amino acids				
Arg	0.33	1.07	0.44	0.01
His	0.21	0.39	0.18	ND
Ile	0.25	0.71	0.22	0.01
Leu	0.84	1.22	0.44	0.03
Lys	0.26	1.00	0.31	0.02
Met	0.13	0.19	0.09	ND
Phe	0.35	0.81	0.28	0.02
Thr	0.26	0.60	0.22	0.01
Trp	0.06	0.29	0.09	< 0.03
Val	0.33	0.71	0.31	0.01
Dispensable amino acids				
Ala	0.51	0.68	0.33	0.02
Asp	0.47	1.71	0.47	0.02
Cys	0.15	0.20	0.13	ND
Glu	1.26	2.81	1.28	0.04
Gly	0.28	0.66	0.36	0.01
Ser	0.32	0.71	0.27	0.01
Tyr	0.19	0.52	0.16	0.01

ND, not detectable.

**Table 4:** Apparent ileal digestibility (%) of amino acids in corn without or with 40 mL of 3 *N* HCl in the collection bag or the collection container

Item (%)	HCl in bag	−		+		SEM	p-value		
		
	HCl in container	−	+	−	+		Bag	Container	Bag×container
Observations (n)		8	8	9	9	-	-	-	-
Indispensable amino acids									
Arg		72.8	73.0	71.0	72.3	6.0	0.875	0.829	0.872
His		79.9	76.6	81.9	81.9	2.5	0.259	0.399	0.384
Ile		73.9	73.5	76.0	77.9	2.2	0.282	0.648	0.463
Leu		84.6	84.1	85.9	86.6	1.4	0.307	0.931	0.565
Lys		70.0	72.8	70.2	72.7	3.3	0.985	0.251	0.951
Met		83.0	83.3	84.8	86.4	1.4	0.179	0.375	0.529
Phe		79.8	79.3	81.5	82.5	1.7	0.242	0.839	0.593
Thr		62.6	61.7	65.1	67.4	3.0	0.305	0.765	0.466
Trp		74.5	74.6	75.3	76.7	2.3	0.616	0.646	0.721
Val		70.7	70.4	73.7	75.1	2.5	0.278	0.724	0.585
Total		76.5	76.2	77.9	79.1	2.1	0.452	0.723	0.568
Dispensable amino acids									
Ala		75.6	76.5	76.9	78.7	2.0	0.492	0.355	0.771
Asp		69.8	69.7	71.3	72.9	2.4	0.471	0.672	0.623
Cys		71.0	71.1	73.6	74.7	2.3	0.317	0.723	0.752
Glu		81.9	82.0	83.5	84.3	1.5	0.336	0.651	0.750
Gly		24.4	29.4	26.1	25.6	8.8	0.929	0.595	0.523
Ser		73.3	73.0	75.0	75.7	2.0	0.409	0.876	0.742
Tyr		71.3	71.3	74.5	75.1	2.5	0.292	0.862	0.868
Total		56.5	60.6	58.7	57.7	4.9	0.960	0.534	0.326
Total amino acids		66.5	68.4	68.6	68.5	3.3	0.821	0.642	0.608

SEM, standard error of the means.

**Table 5:** Apparent ileal digestibility (%) of amino acids in soybean meal without or with 40 mL of 3 *N* HCl in the collection bag or the collection container

Item (%)	HCl in bag	−		+		SEM	p-value		
		
	HCl in container	−	+	−	+		Bag	Container	Bag×container
Observations (n)		9	8	9	9	-	-	-	-
Indispensable amino acids									
Arg		91.7	91.1	89.1	89.8	1.1	0.220	0.990	0.247
His		84.9	86.1	84.6	85.1	1.5	0.742	0.338	0.648
Ile		81.8	82.5	82.8	84.1	1.8	0.618	0.239	0.731
Leu		82.2	82.6	82.8	84.0	1.8	0.703	0.378	0.660
Lys		83.4	84.4	84.7	86.1	1.6	0.515	0.171	0.842
Met		84.4	85.4	84.6	86.5	1.6	0.784	0.060	0.533
Phe		82.4	82.9	83.2	84.3	1.8	0.685	0.345	0.713
Thr		75.6	74.7	75.4	76.5	2.2	0.792	0.934	0.456
Trp		88.8	88.4	88.5	89.1	1.2	0.912	0.856	0.498
Val		77.6	77.5	77.9	79.8	2.5	0.723	0.470	0.459
Total		83.3	83.5	83.3	84.5	1.5	0.807	0.388	0.565
Dispensable amino acids									
Ala		72.3	73.6	73.9	76.7	3.0	0.573	0.216	0.626
Asp		78.2	79.7	80.4	81.1	1.9	0.499	0.357	0.719
Cys		68.9	69.6	70.4	71.1	3.2	0.736	0.714	0.998
Glu		79.5	81.8	82.1	82.6	2.3	0.586	0.310	0.538
Gly		55.1	57.1	59.6	62.8	4.3	0.375	0.332	0.818
Ser		82.8	80.6	81.0	82.0	1.1	0.872	0.487	0.079
Tyr		83.8	82.9	83.3	84.2	1.8	0.878	0.983	0.320
Total		71.3	73.2	73.1	74.8	2.4	0.589	0.232	0.948
Total amino acids		77.3	78.4	78.3	79.7	1.8	0.620	0.272	0.884

SEM, standard error of the means.

**Table 6:** Apparent ileal digestibility (%) of amino acids in wheat middlings without or with 40 mL of 3 *N* HCl in the collection bag or the collection container

Item (%)	HCl in bag	−		+		SEM	p-value		
		
	HCl in container	−	+	−	+		Bag	Container	Bag×container
Observations (n)		8	9	8	9	-	-	-	-
Indispensable amino acids									
Arg		71.2	72.4	67.3	76.3	6.1	0.999	0.245	0.370
His		70.0	72.4	75.1	76.2	3.2	0.316	0.374	0.746
Ile		62.6	63.3	66.7	66.2	2.7	0.239	0.983	0.815
Leu		67.9	68.3	72.5	71.9	2.2	0.083	0.976	0.826
Lys		68.9	69.5	70.4	70.5	3.0	0.744	0.846	0.905
Met		70.2	69.9	76.2	75.4	2.1	0.049	0.741	0.917
Phe		68.3	68.1	72.5	72.1	2.2	0.085	0.878	0.966
Thr		48.7	48.5	54.4	52.5	4.5	0.293	0.817	0.855
Trp		68.6	69.2	70.9	73.7	3.3	0.377	0.592	0.719
Val		60.6	61.7	65.6	65.1	2.6	0.131	0.912	0.754
Total		65.9	66.3	68.6	69.9	2.8	0.338	0.730	0.872
Dispensable amino acids									
Ala		44.3	50.4	47.8	55.3	5.0	0.450	0.148	0.885
Asp		60.3	60.5	61.6	63.5	3.3	0.539	0.748	0.784
Cys		64.5	64.9	66.9	68.5	2.9	0.361	0.734	0.838
Glu		79.8	80.3	80.6	82.1	1.5	0.437	0.483	0.727
Gly		3.0	36.3	3.8	25.5	16.1	0.761	0.106	0.723
Ser		61.1	61.0	63.3	64.2	3.7	0.505	0.911	0.883
Tyr		64.7	64.7	67.5	67.0	3.0	0.435	0.931	0.924
Total		27.1	41.9	19.4	42.6	12.8	0.830	0.049	0.644
Total amino acids		45.9	54.3	42.8	56.0	8.0	0.949	0.078	0.680

SEM, standard error of the means.

**Table 7:** Basal endogenous losses of amino acids (g/kg dry matter intake) without or with 40 mL of 3 *N* HCl in the collection bag or the collection container

Item	HCl in bag	−		+		SEM	p-value		
		
	HCl in container	−	+	−	+		Bag	Container	Bag×container
Observations (n)		9	9	8	9	-	-	-	-
Indispensable amino acids									
Arg		0.60	0.45	0.86	0.86	0.12	0.120	0.290	0.266
His		0.15	0.12	0.13	0.14	0.02	0.965	0.661	0.271
Ile		0.25	0.22	0.17	0.23	0.04	0.440	0.593	0.100
Leu		0.40	0.35	0.28	0.37	0.06	0.516	0.640	0.106
Lys		0.30	0.25	0.18	0.26	0.05	0.411	0.612	0.066
Met		0.06	0.05	0.03	0.05	0.01	0.431	0.778	0.170
Phe		0.26	0.23	0.19	0.24	0.04	0.513	0.644	0.124
Thr		0.47	0.42	0.33	0.44	0.07	0.518	0.453	0.103
Trp		0.08	0.07	0.07	0.08	0.01	0.880	0.749	0.140
Val		0.35	0.31	0.24	0.32	0.05	0.508	0.588	0.149
Total		2.92	2.48	2.41	2.99	0.36	0.994	0.783	0.062
Dispensable amino acids									
Ala		0.60	0.50	0.51	0.64	0.08	0.806	0.839	0.070
Asp		0.61	0.53	0.45	0.59	0.08	0.639	0.615	0.063
Cys		0.15	0.13	0.10	0.15	0.02	0.673	0.460	0.081
Glu		0.78	0.68	0.56	0.71	0.09	0.421	0.737	0.068
Gly		1.81	1.30	1.80	1.98	0.34	0.487	0.340	0.056
Ser		0.44	0.37	0.34	0.43	0.06	0.840	0.741	0.084
Tyr		0.21	0.19	0.16	0.20	0.03	0.593	0.558	0.088
Total		11.18	8.71	13.41	14.33	1.99	0.191	0.491	0.149
Total amino acids		14.13	11.23	15.67	17.31	2.10	0.214	0.636	0.098

SEM, standard error of the means.

**Table 8:** Standardized ileal digestibility (%) of amino acids in corn without or with 40 mL of 3 *N* HCl in the collection bag or the collection containerz^1)^

Item (%)	HCl in bag	−		+		SEM	p-value		
		
	HCl in container	−	+	−	+		Bag	Container	Bag×container
Observations (n)		8	8	9	9	-	-	-	-
Indispensable amino acids									
Arg		89.0	85.2	92.5	95.5	6.0	0.410	0.909	0.303
His		86.1	81.6	87.4	87.9	2.5	0.251	0.311	0.210
Ile		83.0	81.5	82.2	86.0	2.2	0.521	0.447	0.099
Leu		88.9	87.8	88.9	90.6	1.4	0.447	0.781	0.214
Lys		80.3	81.5	76.4	81.7	3.3	0.676	0.163	0.353
Met		86.9	86.5	86.9	89.5	1.4	0.392	0.293	0.166
Phe		86.4	85.2	86.3	88.6	1.7	0.422	0.668	0.194
Thr		78.6	76.2	76.6	82.6	3.0	0.575	0.407	0.063
Trp		86.4	84.6	85.2	87.9	2.3	0.723	0.799	0.208
Val		80.1	78.8	80.3	83.8	2.5	0.443	0.498	0.155
Total		85.1	83.5	85.0	88.0	2.1	0.448	0.618	0.112
Dispensable amino acids									
Ala		86.0	85.2	85.8	89.8	2.0	0.408	0.281	0.111
Asp		81.3	79.6	79.8	84.0	2.4	0.654	0.458	0.097
Cys		79.6	78.6	79.5	83.4	2.3	0.439	0.415	0.161
Glu		87.4	86.8	87.5	89.4	1.5	0.509	0.544	0.235
Gly		82.6	71.4	82.0	88.3	8.8	0.515	0.567	0.055
Ser		85.4	83.4	84.5	87.7	2.0	0.512	0.674	0.094
Tyr		81.3	80.2	82.1	84.7	2.5	0.419	0.664	0.335
Total		83.2	81.5	89.0	90.9	4.9	0.315	0.976	0.472
Total amino acids		84.6	82.8	88.0	90.2	3.3	0.277	0.932	0.301

SEM, standard error of the means.

1) Within treatment values for standardized ileal digestibility were calculated by correcting apparent ileal digestibility values for basal ileal endogenous losses determined from ileal digesta that was treated or not treated with HCl in a similar way.

**Table 9:** Standardized ileal digestibility (%) of amino acids in soybean meal without or with 40 mL of 3 *N* HCl in the collection bag or the collection container^1)^

Item (%)	HCl in bag	−		+		SEM	p-value		
		
	HCl in container	−	+	−	+		Bag	Container	Bag×container
Observations (n)		9	8	9	9	-	-	-	-
Indispensable amino acids									
Arg		97.0^a^	95.0^b^	96.1^ab^	97.3^ab^	1.1	0.637	0.520	0.010
His		88.5	89.0	87.8	88.4	1.5	0.756	0.465	0.932
Ile		85.2	85.5	85.1	87.2	1.8	0.762	0.172	0.329
Leu		85.4	85.4	85.0	86.9	1.8	0.819	0.295	0.293
Lys		86.2	86.8	86.4	88.5	1.6	0.671	0.122	0.353
Met		87.2	87.7	86.2	88.8	1.6	0.990	0.041	0.150
Phe		85.5	85.6	85.4	87.1	1.8	0.787	0.273	0.355
Thr		82.9	81.4	80.7	83.5	2.2	0.972	0.618	0.101
Trp		91.4	90.6	90.6	91.6	1.2	0.958	0.935	0.264
Val		82.2	81.7	81.2	84.0	2.5	0.851	0.354	0.193
Total		87.2	86.8	86.6	88.5	1.5	0.803	0.331	0.163
Dispensable amino acids									
Ala		80.6	80.5	80.9	85.5	3.0	0.527	0.180	0.161
Asp		81.6	82.6	82.9	84.3	1.9	0.561	0.292	0.863
Cys		75.8	75.6	75.1	78.0	3.2	0.841	0.491	0.433
Glu		82.1	84.0	83.9	85.0	2.3	0.653	0.286	0.761
Gly		81.3^ab^	75.9^b^	84.7^ab^	91.0^a^	4.3	0.116	0.850	0.043
Ser		88.6^a^	85.6^b^	85.6^ab^	87.7^ab^	1.1	0.739	0.630	0.008
Tyr		87.7	86.3	86.2	87.9	1.8	0.989	0.850	0.110
Total		84.6	83.7	88.3	91.4	2.4	0.084	0.469	0.185
Total amino acids		86.1	85.4	87.7	90.3	1.8	0.172	0.422	0.170

SEM, standard error of the means.

1) Within treatment values for standardized ileal digestibility were calculated by correcting apparent ileal digestibility values for basal ileal endogenous losses determined from ileal digesta that was treated or not treated with HCl in a similar way.

a,b Within a row, means without a common superscript differ (p<0.05).

**Table 10:** Standardized ileal digestibility (%) of amino acids in wheat middlings without or with 40 mL of 3 *N* HCl in the collection bag or the collection container^1)^

Item (%)	HCl in bag	−		+		SEM	p-value		
		
	HCl in container	−	+	−	+		Bag	Container	Bag×container
Observations (n)		8	9	8	9	-	-	-	-
Indispensable amino acids									
Arg		84.0	82.0	84.3	94.7	6.1	0.412	0.330	0.161
His		77.6	78.6	81.8	83.5	3.2	0.307	0.497	0.861
Ile		73.5	72.9	74.1	75.9	2.7	0.530	0.814	0.649
Leu		76.5	75.9	78.5	79.9	2.2	0.195	0.875	0.649
Lys		78.1	77.2	75.8	78.5	3.0	0.909	0.627	0.335
Met		76.1	74.7	79.4	80.2	2.1	0.116	0.865	0.520
Phe		77.0	75.8	78.8	80.1	2.2	0.190	0.977	0.551
Thr		68.7	66.6	68.6	71.5	4.5	0.598	0.933	0.586
Trp		77.0	76.2	77.9	81.5	3.3	0.420	0.650	0.466
Val		71.2	71.2	73.1	74.9	2.6	0.303	0.730	0.730
Total		76.5	75.4	77.4	80.8	2.8	0.333	0.659	0.381
Dispensable amino acids									
Ala		61.4	64.7	62.3	73.3	5.0	0.387	0.128	0.401
Asp		72.5	71.0	70.6	75.3	3.3	0.727	0.616	0.338
Cys		75.0	74.0	74.1	79.0	2.9	0.515	0.502	0.316
Glu		85.5	85.2	84.7	87.3	1.5	0.703	0.413	0.308
Gly		50.8	70.7	49.7	77.0	16.1	0.875	0.160	0.821
Ser		76.3	74.0	75.3	79.3	3.7	0.601	0.805	0.384
Tyr		77.2	75.9	77.0	79.0	3.0	0.655	0.898	0.570
Total		58.7	66.6	55.4	81.8	12.8	0.717	0.070	0.313
Total amino acids		67.6	71.6	66.2	82.1	8.0	0.652	0.102	0.315

SEM, standard error of the means.

1) Within treatment values for standardized ileal digestibility were calculated by correcting apparent ileal digestibility values for basal ileal endogenous losses determined from ileal digesta that was treated or not treated with HCl in a similar way.
